# Avoiding revolving door and homelessness: The need to improve care transition interventions in psychiatry and mental health

**DOI:** 10.3389/fpsyt.2022.1021926

**Published:** 2022-09-26

**Authors:** Joana Bravo, Francisco Lima Buta, Miguel Talina, Amílcar Silva-dos-Santos

**Affiliations:** ^1^Department of Psychiatry, Hospital Vila Franca de Xira, Vila Franca de Xira, Portugal; ^2^NOVA Medical School, NOVA University of Lisbon, Lisboa, Portugal; ^3^Hospital CUF Tejo, Lisbon, Portugal

**Keywords:** care transition, revolving door, homelessness, rehospitalization, Marontology

## Introduction

In this article, we highlight the need to implement care transition interventions to reduce the revolving door phenomenon (RD) in the general population and homeless patients (HP). We have looked at studies concerning: (1) RD, (2), its impact on HP, and (3) models of care transition interventions in psychiatry and mental health. We conclude with suggestions on improving care transitions in mental health and reducing the RD.

## The revolving door phenomenon

Early hospital readmission is a problem worldwide and an adverse clinical care outcome (1–3). It is estimated to cost $17 billion yearly in the United States (US) (4). In high-income countries, 13% of psychiatric patients are readmitted after hospital discharge (5). In addition, 50% of all discharged psychiatric patients are readmitted within 1 year (6).

The term revolving door means multiple readmissions in a period of 30, 60, or 90 days, according to different studies (7). These patients consume up to 30% of health care resources, although they represent only 10% of the total number of patients (8).

The main factors linked to the RD phenomenon remain uncertain (9). Studies found that revolving door patients are younger, single, with low education and unemployed. They often suffer from psychosis and alcohol or other substance use (10, 11). Also, they have a younger age on disease onset, poor compliance to medication, more suicide attempts and voluntary admissions (12).

There are several reasons for the increase in hospital readmission. One of the main factors is the lack of support from the patient's environment or care system (13). Another cause is early patient discharge before reaching clinical remission and no coordination of medication with the patient or family. Also, a lack of care transition planning and adequate communication among hospital staff, patients, family and primary care providers worsen outcomes (14).

Several treatment strategies reduce hospital readmission: (1) the use of long-acting injectable antipsychotics (15, 16), (2) maintenance electroconvulsive therapy (ECT) (17, 18), and (3) community-based interventions (discussed below). Patients undergoing ECT (19, 20) need a family member or caregiver to monitor them for 24 h after each session. Those without family or social support are vulnerable to RD. To solve this problem, in our department, they are admitted the day before the procedure. They undergo ECT and are discharged after 24 h. A care transition element will follow them up *via* telephone, reminding them of their schedule to ensure they do not miss their maintenance ECT.

### The revolving door problem in the homeless population

Homeless people are a vulnerable population and the RD is especially high among them. They have more medical comorbidities and more mental health problems. A reason for these issues might be their lifestyle. They have more difficult access to health care in the community and do not receive adequate medical care (11, 21).

A large study across three US examined the association of homelessness with hospital readmissions. The four most common causes were: (1) mental illness, (2) peripartum complications, (3) cardiac diseases, and (4) diseases of the digestive system (21).

A study in Nicaragua focused on the gender issue in homelessness. The female population is in a particularly vulnerable position. The “revolving door to homelessness” is more prevalent since they spend multiple episodes living as homeless after having access to independent housing. Also, they had more barriers to finding regular work (22).

It was also shown that men remained homeless for longer periods. A larger proportion of them had alcohol use issues. They also spent time in prison. Women were more prone to use a regular place to spend the night. An important proportion of them suffered sexual violence as a minor. They also suffered intimate partner violence and physical violence as adults. Homelessness in women poses another problem. The children under their care have an increased risk of suffering sexual, physical, or verbal violence (22).

One-fourth to one-third of homeless persons have a severe mental illness. Schizophrenia, bipolar disorder, or major depression are the most prevalent conditions (23). The cost of hospital admissions for the homeless is much higher than in the general population. Homeless people with mental health problems are more likely to use acute and emergency services. Also, they are less likely to receive general primary care than other populations (24).

Mental illness is an independent risk factor for homelessness. Single adults with a major mental illness have a 25–50% risk of homelessness over their lifetime (10, 23). When homelessness and mental illness are combined, the burden on the health system increases. This results in four times higher use of the health services than the housed population (11). Homeless people display low access to community-based health services. Despite being a vulnerable population (with higher illness severity and a higher need for care continuity), they have poor care after discharge (11, 25).

Some authors proposed that homeless people with mental illness should become the object of Marontology. This term origins in the greek word *marontos* which means unwanted. This proposed field is an effort to provide a better response to the particular challenges of this population (26). Other authors suggested that a street Psychiatry rotation should be part of the residency in Psychiatry (27).

### General models of care transition intervention

One of the better-known intervention models in general Medicine is the Care Transitions Intervention created by Eric Coleman and his team (28). It consists of enabling the patient and family to become independent by providing them with the tools and information required. It uses a transition coach that interacts with the patient and family and is based on four pillars: medication self-management, patient-centred record, follow-up and red flags. The transition coach visits the patient in the hospital and home after discharge. Later he follows-up the patient *via* telephone call for 28 days. According to this randomized controlled trial, this intervention reduced early readmission from 13.9% in the control group to 8.3% in the intervention group after 180 days (28).

A prospective cohort study centered on a care transition intervention showed a significant readmission rate reduction compared to the control group (20.0 vs. 12.8%, respectively). It was an intervention based on coaching to empower patients to manage their health and improve their communication with their providers. The complete intervention occurs across 30 days and includes a home visit within 3 days, a first telephone call within 7–10 days, and the final telephone call by day 30 (29).

### Some effective care transition interventions in psychiatry and mental health

There is more research on care transition interventions in general Medicine than in psychiatry (30, 31). Nonetheless, several studies have shown the positive impact of a care plan in the transition from acute mental health inpatient to community care (32–34).

According to a systematic review (31), effective interventions in psychiatry include several aspects, namely: (1) pre and post-discharged psychoeducation; (2) timely communication of the discharge plan to the outpatient provider; (3) pre-discharge medication education; (4) telephone follow-up, and (5) a transition manager.

In terms of care transition models in mental health, Ezra Susser's Critical Time Intervention (CTI) studies in New York (35) was one of the first to show long-term impact and be cost-effective in the prevention of homelessness. Each person was assigned to a CTI worker and provided community housing. The worker would give close support and build durable ties between patients and long-term supports (family, caregivers, psychiatrist, general practitioner). It included home visits, accompanying patients to appointments, giving support and advice and mediating conflicts between patients and caregivers.

Other CTI, showed a significant reduction in homelessness and in readmissions (34). This highlights the importance of strategies that include housing stability to reduce revolving door in the HP.

A network-based concept (32) integrates different health care specialists. This includes psychiatrists, specialized nursing staff and psychologists, social workers and pedagogues. An emphasis is given to psychosocial support and psychoeducation. Other features include socio-therapy, visiting care and family support. This program also includes specialist nursing to provide home treatment. There is cooperation with the hospital in case of admission. Crisis service is available 24/7 for patient and family. The psychiatrist is in charge of the therapy and is the preferred contact for the patient.

Another intervention showed improvements in mental and physical health status, substance misuse, and the number of hospital admissions. It offered case management, peer support, access to primary psychiatric care, and community services (33). Several studies have shown the positive impact of a care plan in the transition from acute mental health units to community care (32–34).

## Discussion

The aggregate data suggest that much more studies about care transition in psychiatry should be conducted. Another note is that the revolving door phenomenon and homelessness remain marginalized. As improvement suggestions, we highlight the need to foster the teaching of care transitions approaches in the residency program of Psychiatry. The development of the subspecialty of Marontology should be considered to address the super difficult patients, revolving door and homelessness (36). There is much more to be done by the mental health services, institutions and the government. Integrative perspectives are relevant to a better knowledge of the mechanisms of mental illnesses (37). This approach merges the knowledge of different areas such as psychiatry and neuroscience, psychology, neuroimaging, and neurology, to name a few. The concept of care transition can be added to the integrative perspectives of mental illnesses.

Important measures of care transition include early follow-up consultation *via* telephone and home visits, psychoeducation, access to prescribed medications, accompanying appointments and bridging ties between patients and long-term supports, such as family members and medical professionals. In order to improve care transition, some initiatives to improve post-discharge outcomes should be encouraged. Care after discharge should be integrative and multidisciplinary.

A particular intervention for the homeless population that includes housing and social support is needed. These measures are cost-effective and have a significant impact in reducing hospital readmission.

## Author contributions

JB and AS-d-S: writing, editing, and review. FB and MT: editing and review. All authors contributed to the article and approved the submitted version.

## Conflict of interest

The authors declare that the research was conducted in the absence of any commercial or financial relationships that could be construed as a potential conflict of interest.

## Publisher's note

All claims expressed in this article are solely those of the authors and do not necessarily represent those of their affiliated organizations, or those of the publisher, the editors and the reviewers. Any product that may be evaluated in this article, or claim that may be made by its manufacturer, is not guaranteed or endorsed by the publisher.
